# Allelic Variants in *Arhgef11* via the Rho-Rock Pathway Are Linked to Epithelial–Mesenchymal Transition and Contributes to Kidney Injury in the Dahl Salt-Sensitive Rat

**DOI:** 10.1371/journal.pone.0132553

**Published:** 2015-07-14

**Authors:** Zhen Jia, Ashley C. Johnson, Xuexiang Wang, Zibiao Guo, Albert W. Dreisbach, Jack R. Lewin, Patrick B. Kyle, Michael R. Garrett

**Affiliations:** 1 Department of Pharmacology and Toxicology, University of Mississippi Medical Center, Jackson, MS, United States of America; 2 Department of Medicine (Nephrology), University of Mississippi Medical Center, Jackson, MS, United States of America; 3 Molecular and Genomics Core Facility, University of Mississippi Medical Center, Jackson, MS, United States of America; 4 Department of Pathology, University of Mississippi Medical Center, Jackson, MS, United States of America; Fondazione IRCCS Ospedale Maggiore Policlinico & Fondazione D’Amico per la Ricerca sulle Malattie Renali, ITALY

## Abstract

Previously, genetic analyses identified that variants in *Arhgef11* may influence kidney injury in the Dahl salt-sensitive (S) rat, a model of hypertensive chronic kidney disease. To understand the potential mechanism by which altered expression and/or protein differences in *Arhgef11* could play a role in kidney injury, stably transduced *Arhgef11* knockdown cell lines as well as primary cultures of proximal tubule cells were studied. Genetic knockdown of *Arhgef11* in HEK293 and NRK resulted in reduced RhoA activity, decreased activation of Rho-ROCK pathway, and less stress fiber formation versus control, similar to what was observed by pharmacological inhibition (fasudil). Primary proximal tubule cells (PTC) cultured from the S exhibited increased expression of *Arhgef11*, increased RhoA activity, and up regulation of Rho-ROCK signaling compared to control (small congenic). The cells were also more prone (versus control) to TGFβ-1 induced epithelial-mesenchymal transition (EMT), a hallmark feature of the development of renal interstitial fibrosis, and characterized by development of spindle shape morphology, gene expression changes in EMT markers (*Col1a3*, *Mmp9*, *Bmp7*, and *Ocln*) and increased expression of N-Cadherin and Vimentin. S derived PTC demonstrated a decreased ability to uptake FITC-albumin compared to the small congenic *in vitro*, which was confirmed by assessment of albumin re-uptake *in vivo* by infusion of FITC-albumin and immunofluorescence imaging. In summary, these studies suggest that genetic variants in the S form of *Arhgef11* via increased expression and/or protein activity play a role in promoting kidney injury in the S rat through changes in cell morphology (Rho-Rock and/or EMT) that impact the function of tubule cells.

## Introduction

Chronic kidney disease (CKD) is seen in all age groups and impacts an estimated 20–30 million people in the United States alone, with hypertension being a major risk factor [1]. Those with CKD initially demonstrate some sign of kidney injury (e.g., proteinuria), but as kidney injury worsens there can be a significant decline in kidney function, leading to an increased risk for renal failure as well as other cardiovascular diseases [2]. The cost associated with treatment of pre-dialysis CKD patients is substantial, with an additional cost associated with dialysis and renal transplantation [3, 4]. Aside from the economic impact, current treatment options are not ideal and only serve to slow the progression of CKD. These factors underscore the importance of identifying genes, pathways and genetic interactions involved in hypertensive related CKD, which could be integral in the development of diagnostic tools and therapeutic targets for prevention and treatment.

Genome wide association studies (GWAS) have been one approach in humans to identify genes/genetic variants associated with complex disease [5]. In particular, there have been a number of large-scale GWAS that have identified single nucleotide polymorphisms associated with kidney injury, but for the most part, these studies have accounted for only a small change in renal injury or a decline in renal function (as reviewed [6]). A second approach has been genetic studies/positional cloning using selectively bred animal models of disease. For example, the Dahl salt-sensitive (S) rat is a widely studied model of hypertension that develops progressive kidney injury characterized by proteinuria, glomerulosclerosis and tubulointerstitial fibrosis, culminating in a significant decline in kidney function [7, 8]. Extensive genetic and genomic analysis has been performed using the hypertensive, but kidney injury resistant spontaneously hypertensive rat (SHR), to identify genetic factors involved in kidney injury [9–13]. At least 9 genomic loci across 8 chromosomes have been linked to proteinuria and/or histological kidney injury in the S rat [13]. A number of gene-gene interactions have also been identified that demonstrated an epistatic effect on kidney injury [13].

The genomic locus on rat chromosome 2 has undergone the most extensive investigation involving several iterations of congenic strain analysis to localize the genomic locus to a small genomic segment. We recently published an extensive analysis of a small congenic strain (with SHR genome substitution on chromosome 2) [9]. Comprehensive analysis using comparative mapping, haplotype analysis, concordance with human genetic studies, sequencing and expression studies, narrowed the locus to <375 kb and identified *Arhgef11* as a strong candidate gene involved in kidney function [9]. ARHGEF11 is a Rho guanine nucleotide exchange factor that participates in the Rho-ROCK pathway through catalyzing the exchange of GDP for GTP to activate RhoA and downstream signaling cascade. ARHGEF11 protein levels and other downstream signaling proteins (RHOA, ROCK1, LIMK1, p-Cofilin) were observed to be significantly elevated in the S kidney compared to the small congenic. The decreased activation of the Rho-ROCK pathway were associated with a significant improvement in proteinuria, tubulointerstitial fibrosis, and kidney function in the small congenic (e.g. RBF, GFR) [9]. While both strains exhibited similar glomerular permeability (i.e. same amount of protein in the filtrate), differences in proteinuria suggested that the reuptake of filtered protein in proximal tubules was linked to the underlying genetic mechanism. Based on this data, we hypothesized that chronic stimulation of the Rho-ROCK pathway in proximal tubules/cells (conferred by genetic variants in S allele of *Arhgef11*) accounts, in part, for progressive kidney injury observed in the S model.

The objective of the current study was to investigate the potential mechanism by which altered expression and/or protein function in ARHGEF11 could play a role in kidney injury exhibited by the Dahl S. We sought to answer several questions, including: what was the influence of genetic knockdown of *Arhgef11* on RhoA activity and Rho-ROCK signaling pathway *in vitro*? How does genetic knockdown of *Arhgef11* compare with downstream inhibition of ROCK using a pharmacological agent? Do primary proximal tubules cells cultured from the S and the small congenic (renal protective) exhibit differences in RhoA activity and Rho-ROCK signaling cascade? What impact, if any, does the pro-inflammatory cytokine TGFβ-1 have on primary proximal tubules cells cultured from these strains? Are there functional differences between proximal tubules cells cultured from both strains and are there *in vivo* implications? In summary, by addressing these questions it is expected that a deeper understanding of role of *Arhgef11* in kidney injury and associated genetic mechanism could lead to a novel diagnostic or therapeutic target for treatment of hypertensive CKD.

## Results

### Molecular and experimental evidence of *Arhgef11* involvement in kidney injury

Previous linkage analyses, congenic strain analysis, and molecular approaches (including sequencing and expression analysis) narrowed a genomic locus on rat chromosome 2 associated with kidney injury exhibited by the Dahl salt-sensitive (S) rat (**S1 Fig**). In particular, the nature and type of sequence variation (coding/promoter) (**S2 Fig**), increased expression, and biological role of *Arhgef11* (via Rho-ROCK pathway) suggested that this gene underlies the genomic locus.

### Physiological impact, kidney localization, and expression of *Arhgef11*

**Fig 1:**
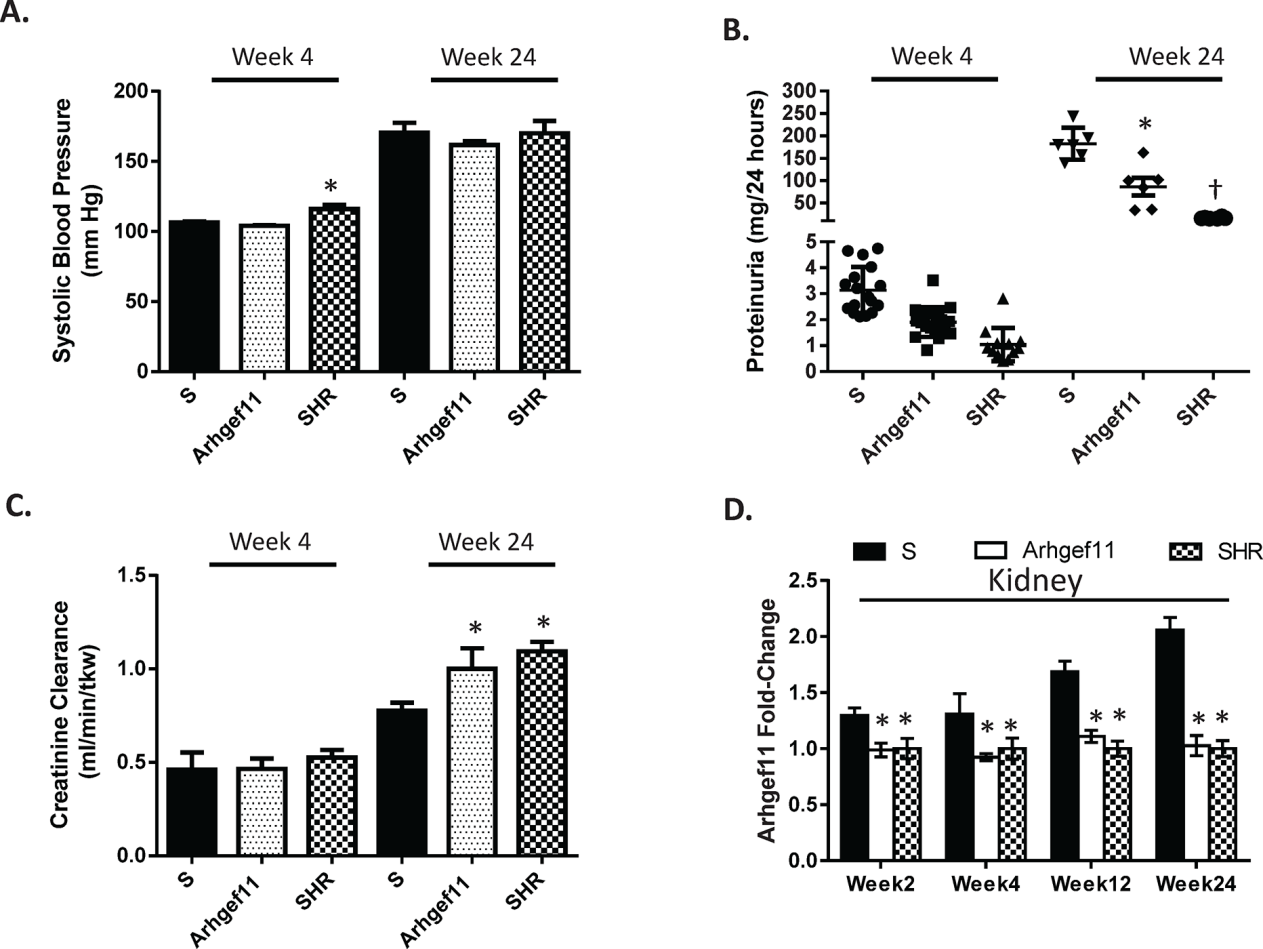
Blood pressure, proteinuria, and renal function measurements and correlation with changes in *Arhgef11* expression. (**A**) Systolic blood pressure in S, Arhgef11-congenic (C), and SHR at week 4 and 24 (n = 6 per group). (**B**) Proteinuria as a measure of renal injury (n = 6–20). (**C**) Creatinine clearance as an indication of renal function (n = 4–6 per group). (**D**) Renal expression of *Arhgef11* from week 2 (before phenotypic differences between strains) until week 24 (n = 6 per group/time). Arhgef11-congenic is genetically similar to the S rat, except for allelic difference in *Arhgef11* (S2 Fig) and few surrounding genes. *p<0.05 versus S, †, p<0.05 versus S and C. SE are presented.

Systolic blood pressure (SBP), proteinuria, and creatinine clearance (CrCl) were evaluated between the S, Arhgef11-congenic, and SHR at week 4 and 24 (**Fig 1A–1C**). At week 4, SBP was not significantly different between the S and Arhgef11-congenic, while the SHR exhibited significantly elevated blood pressure. All three groups demonstrated negligible levels of proteinuria (1–3 mg/24 hours) and no significant difference in renal function (CrCl) at week 4. By week 24, SBP was significantly elevated (~160 mm Hg) compared to week 4, but there was no difference between all three groups. From week 4 to 24, proteinuria in the S rat increased to 182±14.6 mg/24 hours, while the increase in the Arhgef11-congenic was significantly attenuated (86±19.6mg/24 hours) compared to the S. The SHR demonstrated only a small change in proteinuria from week 4 to 24 (16.5±0.8 mg/24 hours), which was well below that exhibited by the S and Arhgef11-congenic. Renal function, by week 24, was significantly decreased in the S (0.7±0.04 ml/min/g kidney weight) compared to the Arhgef11-congenic and SHR (1.0±0.11 and 1.1±0.05, respectively). In the S, a significant increase in *Arhgef11* kidney expression (compared to Arhgef11-congenic and SHR) was observed to precede the development of proteinuria and increased expression correlated with progressive injury and decline in kidney function in the S rat (**Fig 1D**).

**Fig 2:**
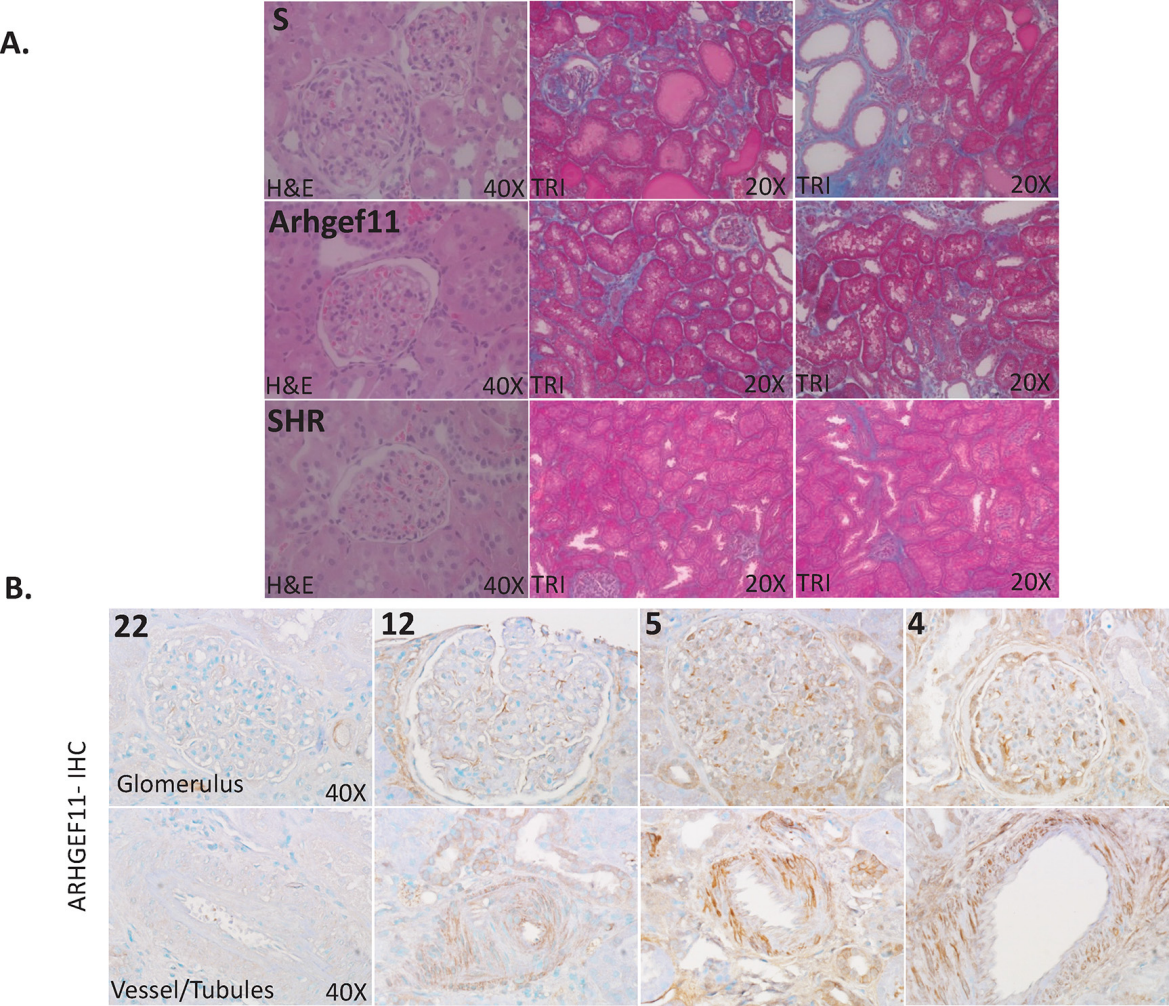
Kidney pathology in the rat and immunohistological staining of ARHGEF11 in human kidney biopsies. (**A**) Representative glomerular and tubulointerstitial images for S, Arhgef11-congenic, and SHR at week 24. The S rat demonstrated glomerular injury (mesangial expansion, glomerulosclerosis, etc), tubular injury, and interstitial fibrosis (tubule atrophy, immune cell infiltration, and/or fibrosis), consistent with detailed histological analysis previously described.[9] Both the Arhgef11-congenic and SHR demonstrated less glomerular and tubular injury compared to S. (**B**) ARHGEF11 staining in human kidney biopsy material from patients with various etiologies, including control (biopsy 22-no specific renal pathology; nephrectomy subsequent to renal pelvic carcinoma); biopsy 12, hematuria and changes compatible with acute tubular necrosis; biopsy 5, hematuria and non-nephrotic range proteinuria; and biopsy 4, diabetic glomerulosclerosis, interstitial fibrosis, and tubular necrosis).A representative image of staining in the glomerulus is shown in the upper panel and vessel and some tubular staining is illustrated in the lower panel.

As expected, there were no significant pathological changes identified in any of the strains at week 4 (data not shown). However, by week 24, the S rat demonstrated significant glomerulosclerosis and tubulointerstitial injury (demonstrated by blue staining), which was significantly attenuated in the Arhgef11-congenic (**Fig 2A**). ARHGEF11 staining in human kidney biopsies from patients with various CKD etiologies demonstrated staining in the glomerulus, tubules, and vessels (**Fig 2B**). The intensity of ARHGEF11 staining appeared to correspond with the degree of injury as increased staining was observed in kidney demonstrating the most severe injury.

### Establishment of stably transduced *Arhgef11* knockdown cells

**Fig 3:**
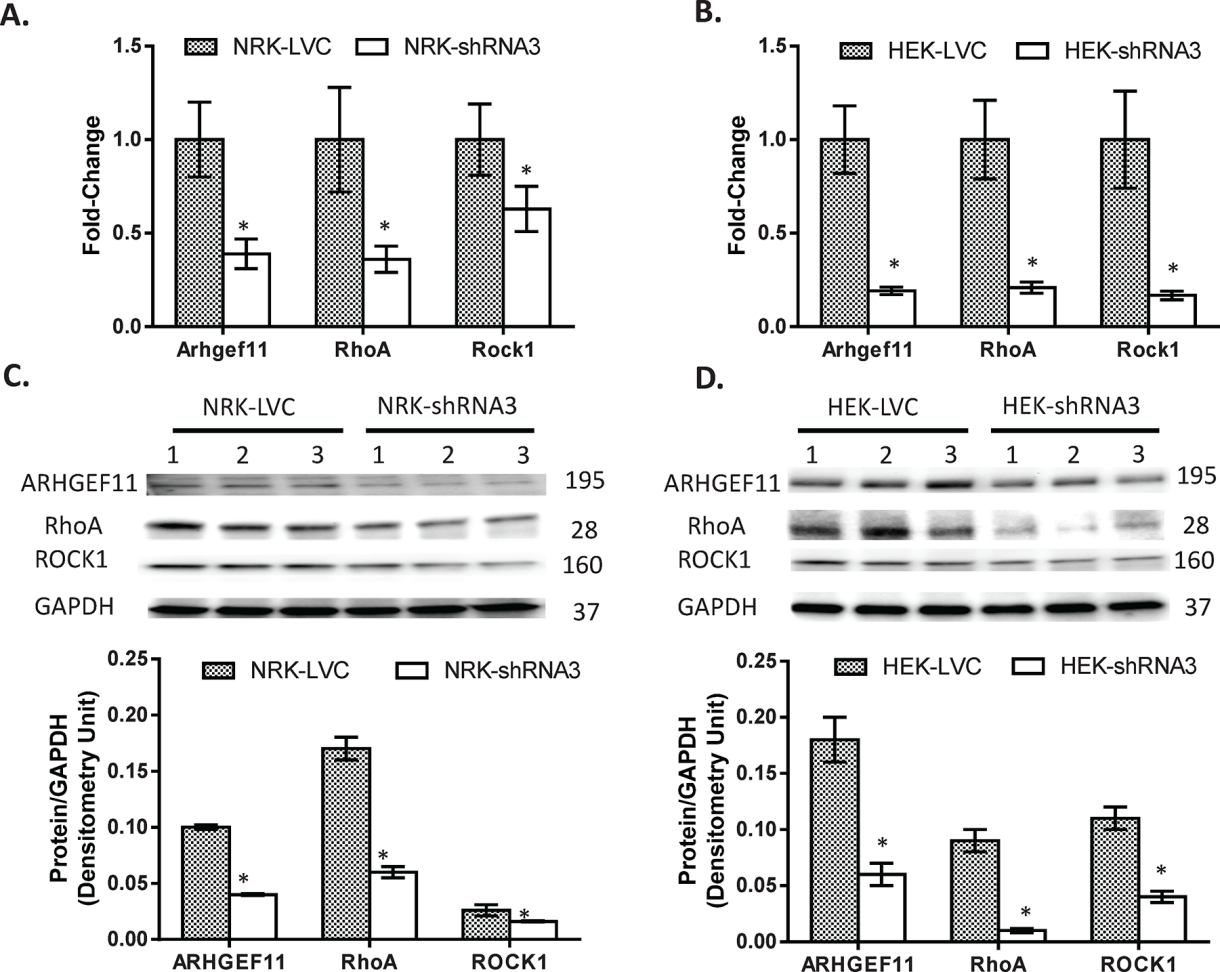
Establishment of stably transduced *Arhgef11* knockdown cell lines using shRNA lentiviral methodology. A number of shRNA lentiviral constructs (shRNA1-4) were tested for optimal knockdown of *Arhgef11* in both NRK and HEK293T cells (see **S2 Fig**). (**A**) Real-time PCR confirmation of shRNA3 *Arhgef11* knockdown and subsequent impact on RhoA and ROCK in NRK stably transduced cells compared to scrambled control (LVC). (**B**) Real-time PCR confirmation of shRNA3 *Arhgef11* knockdown and impact on RhoA and ROCK in HEK293T stably transduced cells compared to scrambled control (LVC). (**C**) Western analysis and densitometry measurements of ARHGEF11, RhoA, and ROCK between NRK-LVC and NRK-shRNA3.(**D**) Western analysis and densitometry measurements of ARHGEF11, RhoA, and ROCK between HEK293T-LVC and HEK293T-shRNA3. n = 4–6 independent samples/group, *p<0.05 versus LVC, Error bar are ±SD.

A number of shRNA lentiviral constructs (1–4) were transduced into NRK and HEK293 cells to identify a cell-line with optimal knockdown of *Arhgef11* (**S3 Fig**). shRNA3 and 4 transduced cells lines demonstrated the most significant decrease in *Arhgef11* (versus scrambled shRNA control) compared to shRNA1 and 2. Given that shRNA3 was a perfect match for both rat and human *Arhgef11*, subsequent studies used shRNA3 transduced cell lines. On average, NRK-shRNA3 transduced cells resulted in ~60% knockdown of *Arhgef11* compared to NRK-LVC (scrambled shRNA control), whereas HEK293-shRNA3 demonstrated slightly greater knockdown (~80%) compared to control (HEK293T-LVC) (**Fig 3A and 3B**). Expression of downstream genes *RhoA* and *Rock1* were also found to be downregulated as a result of reduced *Arhgef11* expression. Protein levels of ARHGEF11, RhoA, and ROCK1 were all significantly (p<0.05) decreased in either NRK/HEK293-shRNA3 compared to LVC (**Fig 3C and 3D**).

### Genetic knockdown of *Arhgef11* on Rho-ROCK pathway versus pharmacological inhibition

**Fig 4:**
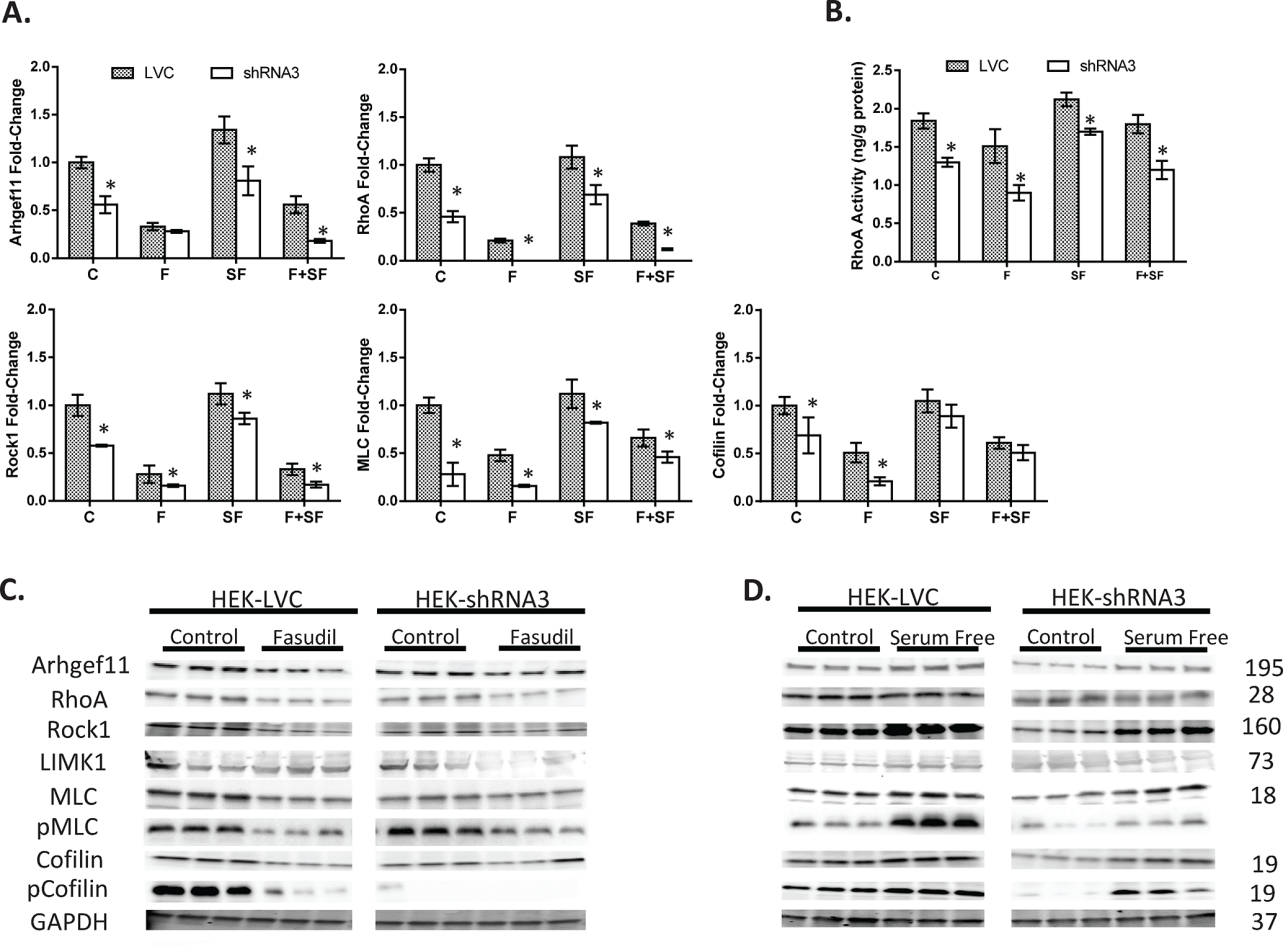
Analysis of the Rho-ROCK signaling pathway and RhoA activity in stably transduced *Arhgef11* knockdown cell lines. Cell-culture experiments were performed under several experimental conditions: 1) control (DMEM medium); 2) fasudil (F) (10μg/ml) treated for 4hrs; 3) serum free (SF) for 24 hours, or 4) serum free for 24 hrs + fasudil treated for 4 hours (SF+F). (**A**) Real time PCR of *Arhgef11*, *RhoA*, *Rock1*, *MLC* and *Cofilin* of HEK293-shRNA3 compared to LVC (scrambled control). (**B**) RhoA activity between LVC and HEK293-shRNA3 under each experimental condition. (**C**) Western analysis of Rho-Rock pathway in LVC and HEK293-shRNA3 between control and fasudil treatment; and (**D**) Western analysis of Rho-Rock pathway in LVC and HEK293-shRNA3 between control and serum free conditions. Similar results were observed for LVC and NRK-shRNA3 for control versus fasudil (**S3 Fig**). n = 6 independent samples per group/treatment, *p<0.05 versus LVC. Error bars are ±SD.

LV-shRNA3 (and LVC) cells were grown and studied under four experimental conditions: 1) control (C); 2) fasudil (F, an inhibitor of ROCK); 3) serum free (SF); and 4) fasudil +serum free (F+SF). The fasudil group was studied to determine whether knockdown of *Arhgef11* would have a similar impact on the Rho-ROCK pathway compared to pharmacological inhibition. Expression of *Arhgef11* and downstream factors (*RhoA*, *Rock1*, *MLC* and *Cofilin*) were significantly decreased in LV-shRNA3 compared to LVC under control conditions (**Fig 4A, S4 Fig**). Decreased expression of *Arhgef11* was associated with a significant decrease in RhoA activity compared to LVC under control conditions (**Fig 4B, S4 Fig**). Similar to genetic knockdown of *Arhgef11*, fasudil treatment resulted in decreased expression for all genes in RhoA-Rock pathway under control conditions, including genes upstream of ROCK (presumably due to a feedback mechanism related to changes in the cytoskeleton). However, expression of RhoA-Rock pathway genes were down-regulated in LV-shRNA3 +F (knockdown of *Arhgef11* and inhibition of ROCK) more than LVC +F cells (only inhibition of ROCK).

Expression of RhoA-Rock pathway genes demonstrated increased expression under serum-free conditions compared to control conditions (**Fig 4A**). This was consistent with significantly increased RhoA activity in both groups, while LV-shRNA3+SF exhibited less activity compared to LVC+SF. Genetic knockdown of *Arhgef11* under serum free conditions demonstrated similar RhoA activity to levels observed for LVC under control conditions (i.e. RhoA activity in LV-shRNA + SF was normalized to LVC levels observed under control conditions)(**Fig 4B**).

Cells treated with fasudil and under serum free (F+SF) conditions resulted in down regulation of all Rho-ROCK pathway genes compared to control conditions (C) for both LVC and LV-shRNA3, but not to the extent observed with fasudil (F) treatment alone. RhoA activity for both LVC and LV-shRNA3 cells treated with fasudil and under serum free conditions were normalized to control levels (**Fig 4B**). Protein levels of ARHGEF11 and downstream proteins RhoA, ROCK, LIMK, MLC, and Cofilin were consistent with gene expression findings for each cell culture condition and between LCV and *Arhgef11* knockdown cells (**Fig 4C and 4D, S5 Fig**). The phosphorylated form of MLC and cofilin demonstrated the most significant impact on protein levels, regardless of genetic knockdown of *Arhgef11* or fasudil treatment. Protein levels of pMLC and pCofilin in the LV-shRNA3 cells were significantly lower and changed to a less extent compared to LVC (control vs. fasudil). In contrast, protein levels of pMLC and pCofilin in the LV-shRNA3 increased to a greater extent than LVC between control and serum free conditions (**Fig 4C and 4D, S5 Fig**).

### Cellular localization of ARHGEF11 and impact on cytoskeleton

**Fig 5:**
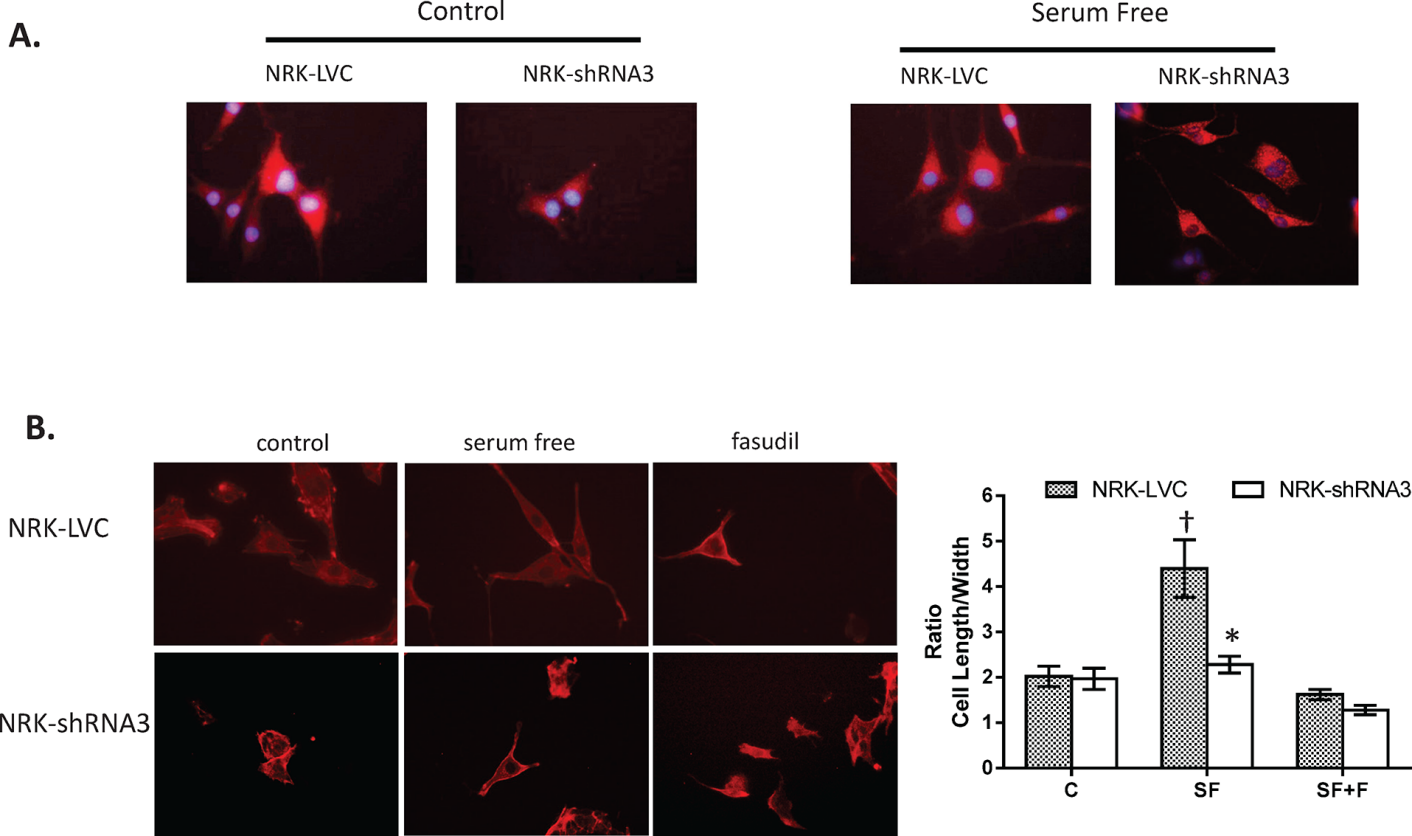
ARHGEF11 localization and actin (F-actin) cytoskeleton immunofluorescence. (**A**) Localization of ARHGEF11 in stably transduced NRK cells [-LVC (lentiviral scramble control) or–shRNA3]. ARHGEF11 is located near the nucleus and throughout the cell under control conditions for both LVC and LVC-shRNA3 cells, Under serum free conditions ARHGEF11 appears to be distributed throughout the cell and toward the cell membrane. (**B**) Immunofluorescence of F-actin in stably transduced NRK cells under control, serum free and fasudil treatment. Cells cultured under serum free conditions develop stress fibers and demonstrate elongation of cells. NRK-shRNA3 cells demonstrate a significant reduction in stress fibers, similar to pharmacological imbibition of ROCK by fasudil, as cell length/width ratio decreases to baseline with genetic knockdown of *Arhgef11*. *p<0.05, n = 3 independent samples/20 random images per slide (6–10 cells per view). Error bars are ±SD.

ARHGEF11 localized throughout the cell, including the nucleus under control conditions for both LVC and *Arhgef11* genetic knockdown cells (LV-shRNA3), while LV-shRNA3 cells demonstrated less fluorescence and regions of punctate staining (**Fig 5A**). The distribution of ARHGEF11 under serum free conditions appeared to be more cytosolic/membrane and less nuclear, but this was more obvious in LV-shRNA3 cells due to less staining of ARHGEF11. Cytoskeletal F-actin immunofluorescence under control conditions showed that both control and *Arhgef11* knockdown cells exhibited similar morphology (**Fig 5B and 5C**) However, under serum free conditions, LVC cells exhibited ~2-fold increase in cell elongation (and stress-fibers), significantly more than that observed in LV-shRNA3 cells (**Fig 5B and 5C**). Similar to *Arhgef11* knockdown cells, LVC cells treated with fasudil demonstrated morphology similar to control conditions.

### Cultured primary proximal tubules and susceptibility to TGFß-1 induced epithelial mesenchymal transition (EMT)

**Fig 6:**
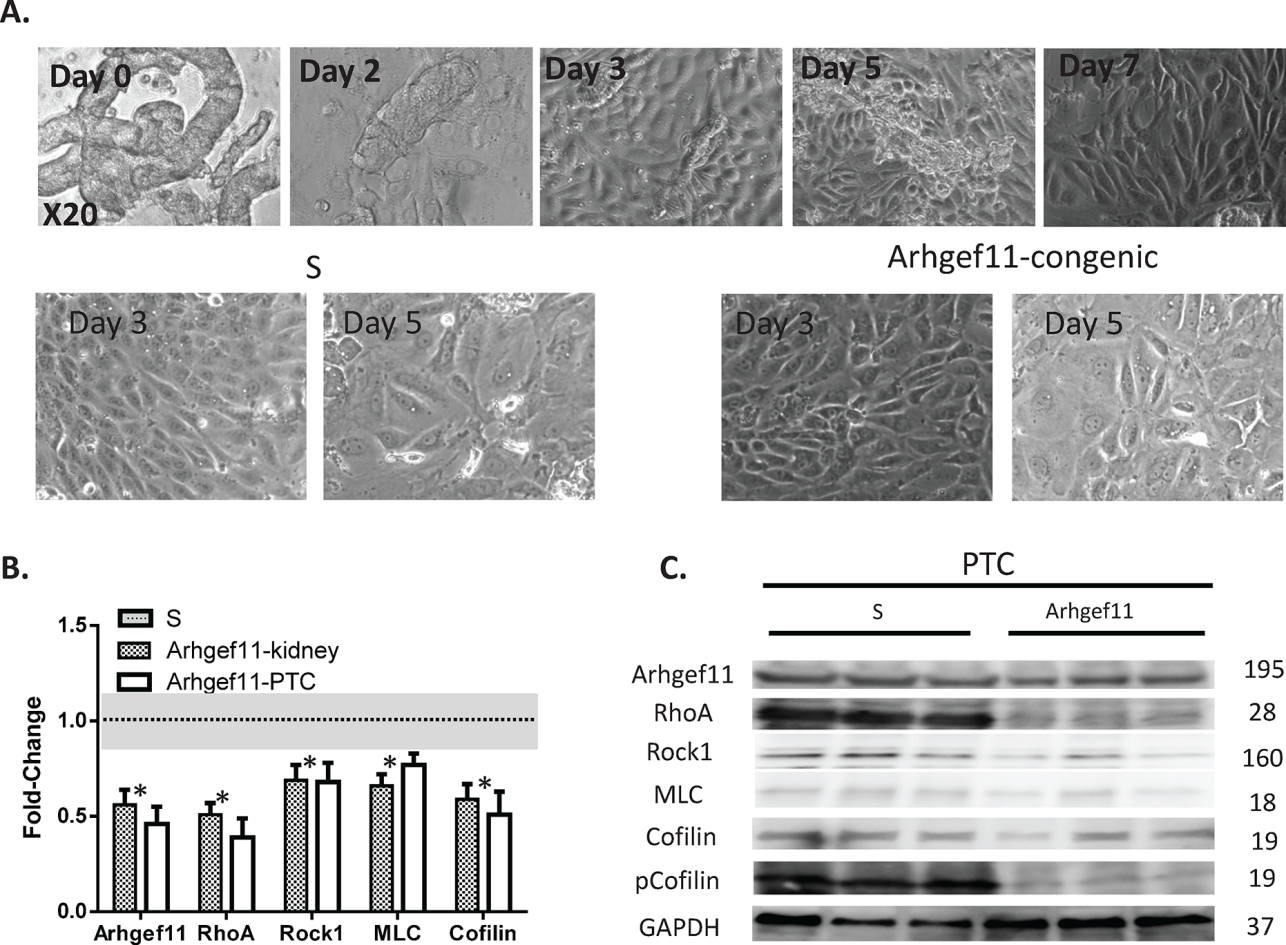
Primary proximal tubule cell (PTC) culture and Arhgef11-Rho-ROCK signaling pathway. (**A**) Representative phase-contrast images (at 20X) of isolated tubular fragments (Day 0) and outgrowth and culture of proximal tubules cells (Day 2–7). There appears no obvious morphological difference between PTC derived S and Arhgef11-congenic. (**B**) Real-time PCR of Arhgef11-Rho-ROCK signaling genes from kidney tissue and PTC acquired from S and Arhgef11-congenic at 4–6 weeks of age. Cultured PTCs (Day 5) exhibit a similar down-regulation of Arhgef11 and signaling pathway as kidney tissue. (**C**) Western analysis of Rho-Rock pathway in cultured PTC. n = 4–6 independent samples, *p<0.05 versus S, Error bar are ±SD.

Kidneys were isolated from S and Arhgef11-congenic animals at 4 weeks of age, well before significant differences in renal injury and renal function were detected between strains (**Fig 1**). Primary proximal tubule cells (pPTC) were grown from tubular fragments isolated from kidneys of both strains (**Fig 6A**). pPTC from both strains exhibited the classic cobblestone-like appearance, with no obvious difference in cell growth or morphology between groups. Expression of *Arhgef11* and *RhoA* was ~50% lower in kidney tissue and pPTC cultured from Arhgef11-congenic compared to S, while *Rock1*, *Mlc*, and *cofilin* exhibited a smaller (~25–50%), but significant difference (**Fig 6B**). Protein levels of ARHGEF11 and downstream proteins of Rho-ROCK were consistent with gene expression findings (**Fig 6C, S6 Fig**). Similar to LV-shRNA cells, phosphorylated MLC and Cofilin demonstrated the most significant impact on protein levels between the S and Arhgef11-congenic pPTC. RhoA activity was significantly lower in Arhgef11-congenic pPTC compared to S (**S6 Fig**)

**Fig 7:**
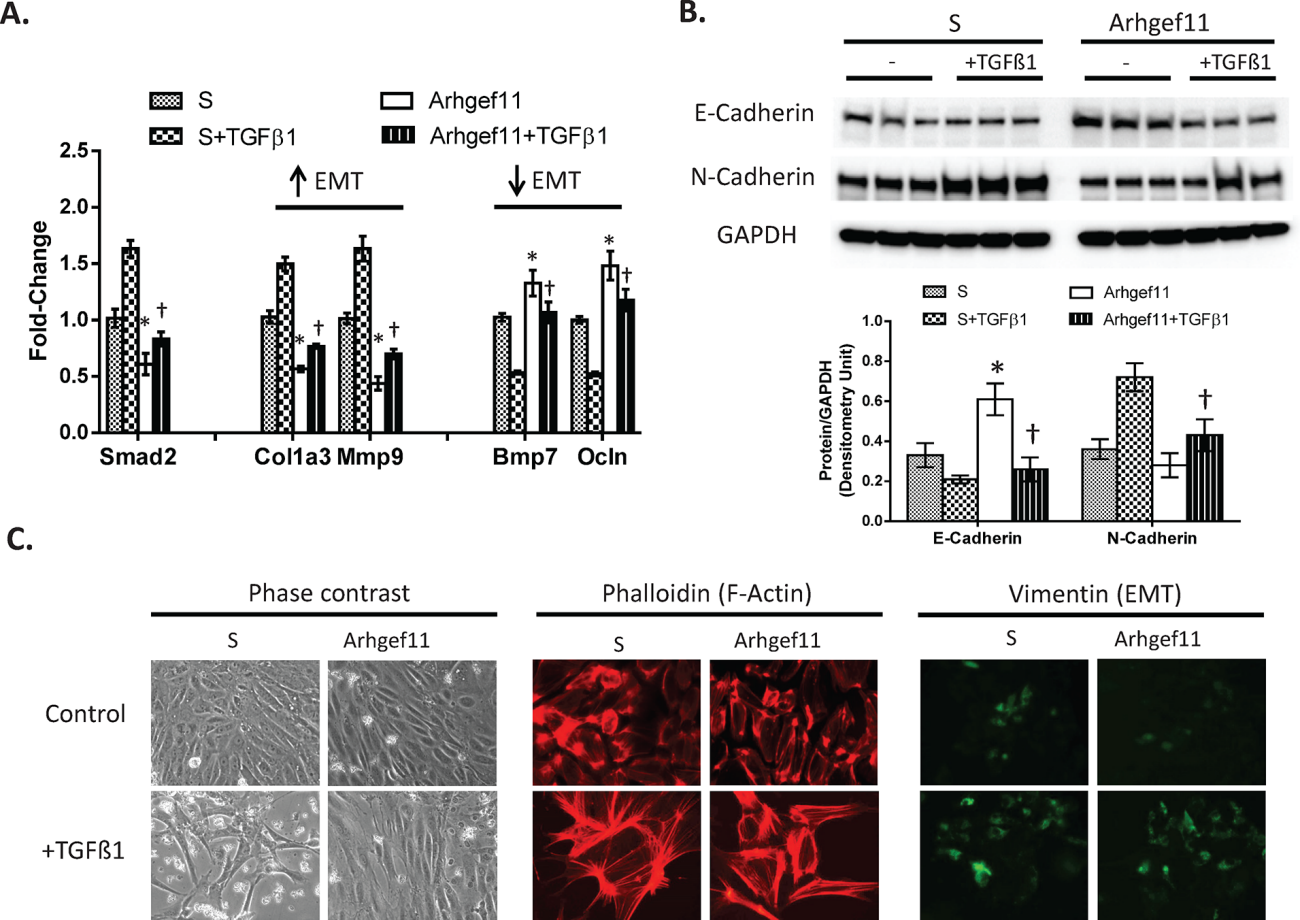
Real-time PCR and western analysis of TGFß-1 induced epithelial mesenchymal transition (EMT) and impact on RhoA-Rock pathway in primary proximal tubule cell (PTC). Primary PTC cells (day 5, >60% confluent) were grown from S and Arhgef11-congenic kidney (at 4 weeks of age) and treated with TGFß-1 (10 ng/ml) for 48 hrs. (**A**) Real-time PCR of markers indicative of EMT. (**B**) Western analysis and densitometry of E-Cadherin and N-Cadherin EMT markers. (**C**) Representative phase contrast and immunofluorescence of stress fibers formation (F-Actin) and Vimentin (EMT markers) between S and Arhgef11-congenic PTC exposed to TGFß-1. n = 4–6 independent samples, *p<0.05 versus S, †p<0.05 versus S+ TGFß-1. Error bar are ±SD.

Baseline gene expression levels of epithelial mesenchymal transition (EMT) markers (*Col1a3*, *Mmp9*, *Bmp7*, and *Ocln*) were significantly different between S and Arhgef11-congenic derived pPTC (**Fig 7A**). Specifically, *Col1a3* and *Mmp9* were increased >50% in S pPTC (increased in EMT) whereas *Bmp7* and *Ocln* were decreased in S pPTC ~50% (decreased in EMT) compared to Arhgef11-congenic derived pPTC. Under baseline conditions, protein levels of E-Cadherin were significantly elevated (50%) and N-Cadherin significantly decreased (10%) in Arhgef11-congenic compared to S pPTC (**Fig 7B**). Treatment with TGFß-1 resulted in increased expression of *Col1a3* and *Mmp9* over baseline for both S and Arhgef11-congenic pPTC, while expression was significantly attenuated in Arhgef11-congenic compared to S pPTC. TGFß-1 treatment led to decreased expression of *Bmp7* and *Ocln* in S pPTC to a greater extent compared to Arhgef11-congenic pPTC (**Fig 7A**). With TGFß-1, protein levels of E-Cadherin were significantly decreased (50%) and N-Cadherin increased (2-fold) in S pPTC compared to baseline (**Fig 7B**). However, there was no dramatic change in E-Cadherin and N-Cadherin between Arhgef11 pPTC and those treated with TGFß-1. RhoA activity was significantly increased over control in both S and Arhgef11 pPTC treated with TGFß-1, but RhoA activity was significantly decreased in Arhgef11+ TGFß-1 pPTC compared to S+ TGFß-1 (**S6 Fig**).

Under baseline conditions, light microscopy morphology of S and Arhgef11-congenic pPTC appeared similar, while S pPTC demonstrated more F-actin and Vimentin immunofluorescence compared to Arhgef11-congenic pPTC (**Fig 7C)**. TGFß-1 treatment led to a dramatic change in cell morphology in S pPTC with enhanced immunofluorescence in F-actin and Vimentin compared to Arhgef11-congenic pPTC.

### Functional evaluation of primary proximal tubule cell (pPTC) and *in vivo* confirmation

**Fig 8:**
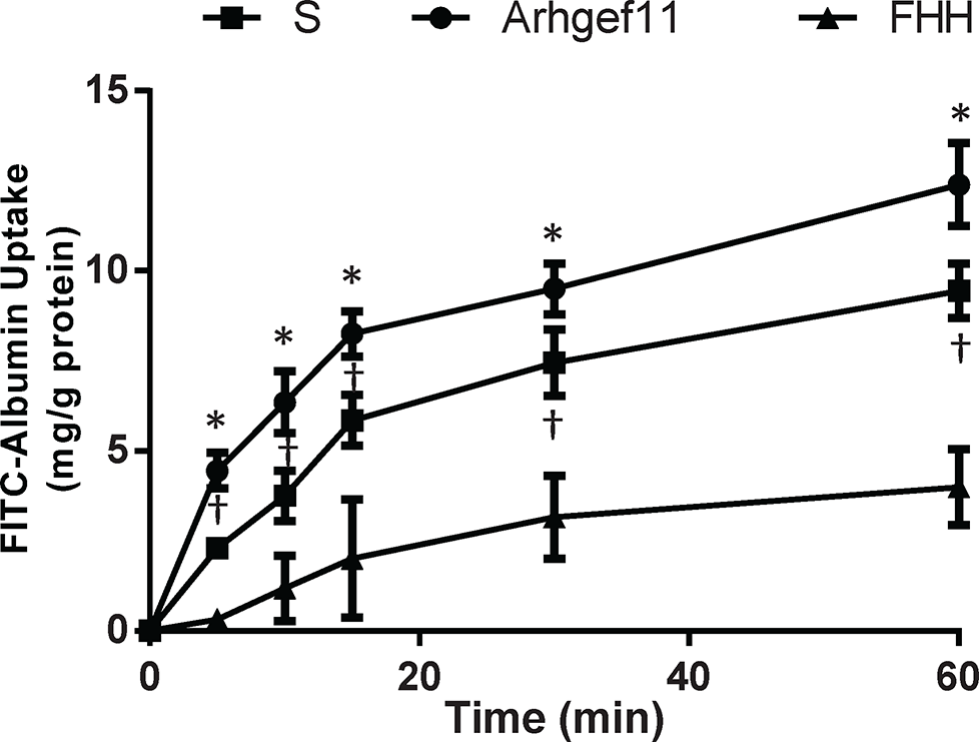
*In vitro* assessment of albumin uptake in primary proximal tubule cell (PTC). Primary PTC cells (day 5, >60% confluent) were grown from S and Arhgef11-congenic kidney (at 4 weeks of age) and incubated with FITC-Albumin (10 ug/ml) at 0, 5, 10, 15, 30, 60 min. Primary PTC were derived from fawn-hooded hypertensive (FHH) rat as comparison due to a known genetic defect that impairs tubular re-uptake of albumin/protein (i.e., low albumin uptake). PTCs from S kidney exhibited decreased uptake of FITC-albumin compared to PTC from Arhgef11-congenic, while FHH demonstrated the least ability uptake FITC-albumin. n = 3–6 independent samples, *p<0.05 versus S and FHH, †p<0.05 versus FHH. Error bars are ±SD.

pPTC grown from the S, Arhgef11-congenic, and a control with a diminished capacity to re-uptake protein [fawn-hooded hypertensive (FHH)], were accessed for ability to uptake FITC-albumin (**Fig 8**). Arhgef11-congenic pPTC demonstrated an enhanced ability (1.5 fold, p<0.05) to uptake FITC-albumin compared to S pPTC, but both S and Arhgef11 pPTC demonstrated a significant increase in uptake of FITC-albumin over defective FHH pPTC (2–2.5 fold, p<0.05).

**Fig 9:**
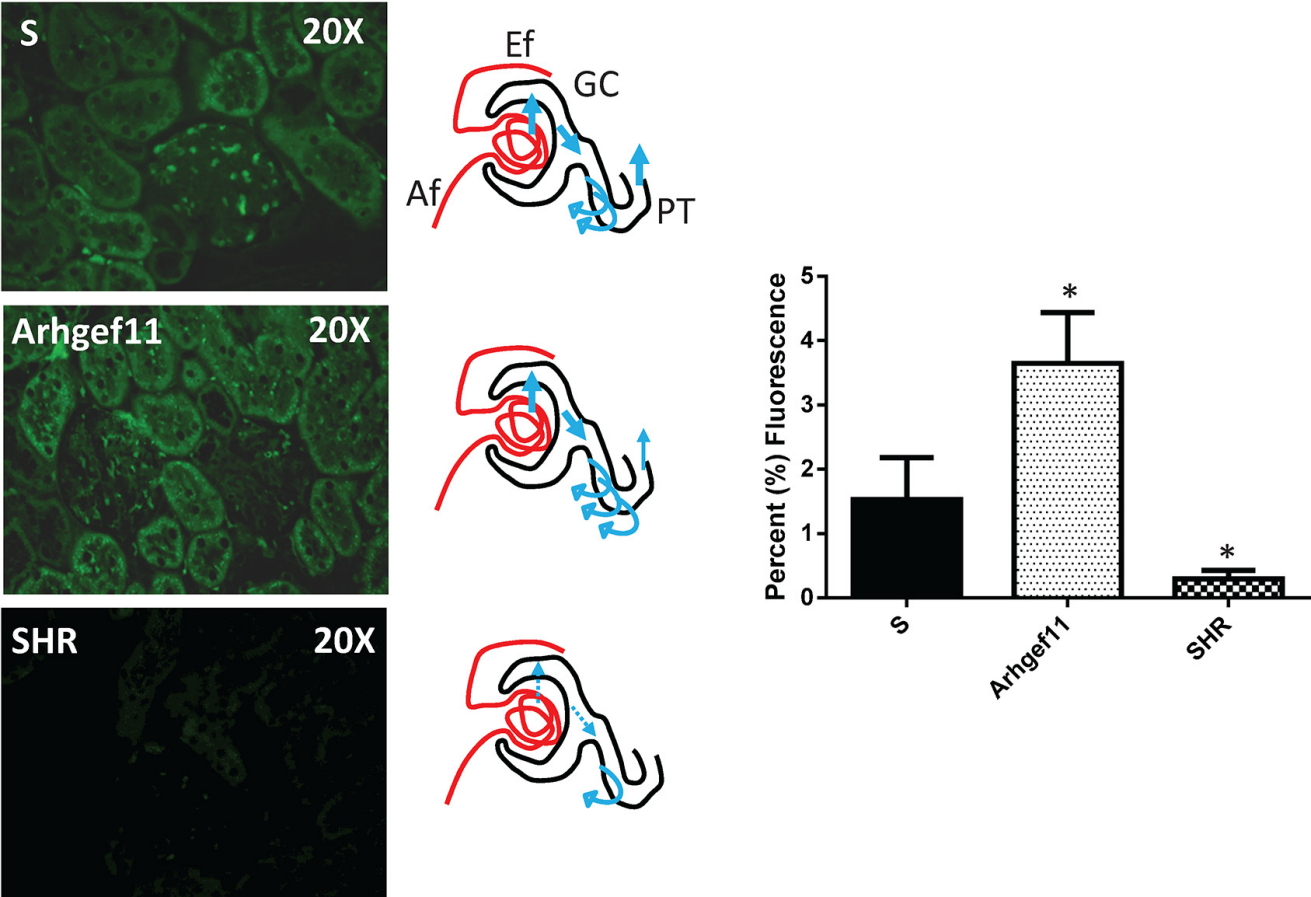
*In vivo* assessment of albumin re-uptake by infusion via FITC-albumin and immunofluorescence imaging. FITC-labeled albumin was infused in S, Arhgef11-congenic and SHR (n = 5–6 per group) at a rate of 100 ul/min for 15 min. Subsequently, kidneys were collected, fixed in a 10% buffered formalin solution, processed and sectioned, and imaged. Threshold image analysis was used to measure the degree of fluorescence in the kidney from each strain (n = 2 sections per kidney and 10–15 images per section). Kidneys from Arhgef11-congenic exhibited an increase in tubular fluorescence/punctate regions of active albumin uptake compared to kidney from S. Fluorescence in SHR kidney was low as little albumin is filtered and remains in glomerular vasculature. Af, afferent arteriole; Ef, efferent arteriole; GC, glomerular capillary, PT, proximal tubule. The thicker the blue arrow the more protein that occurs in the ultrafiltrate and enters the proximal tubule/urine. The number of curved arrows denote active reuptake of filtered protein. *p<0.05 versus S. Error bar are +SE.

Assessment of albumin re-uptake *in vivo* was performed by infusion of FITC-albumin and immunofluorescence imaging (**Fig 9**). FITC-labeled albumin was infused in S, Arhgef11-congenic, and SHR. Kidneys were collected, fixed, processed and sectioned, and evaluated for degree of immunofluorescence. Kidney from Arhgef11-congenic exhibited ~2-fold increase in the intensity of fluorescence in proximal tubules, including more punctate staining within each tubule compared to S kidney. Kidney from SHR demonstrated minimal fluorescence compared to either S or Arhgef11-congenic.

## Discussion

Through positional cloning, *Arhgef11*, a Rho guanine nucleotide exchange factor was previously implicated in kidney injury and reduced function exhibited by the Dahl salt-sensitive (S) rat, a model of hypertensive related chronic kidney disease (CKD) [9, 12]. An extensive characterization of an Arhgef11-congenic strain suggested that the most likely physiological mechanism associated with *Arhgef11* was tubular mediated as glomerular permeability (Palb) and glomerular injury was similar between the two strains [9]. The Arhgef11-congenic exhibited decreased proteinuria, tubulointerstitial injury, and fibrosis, which was associated with improved renal hemodynamics [9]. We also found that i*n vivo* pharmacological inhibition of ROCK with fasudil (downstream from ARHGEF11) improved kidney injury and function in the S rat, but had little impact on the Arhgef11-congenic likely due to an appropriate regulation of the Arhgef11-Rho pathway. The animal studies, in total, suggested that dysregulation of Rho-ROCK (via ARHGEF11) in tubular cells likely play an important role in promoting EMT and fibrosis in the S kidney. In the present study, we sought to investigate the cellular impact of altering expression of *Arhgef11* on Rho-ROCK pathway using genetic knockdown, followed by investigation of the impact of variants in *Arhgef11* on Rho-ROCK using primary proximal tubular cells isolated from the S and the Arhgef11-congenic model.

The main function of ARHGEF11 is to catalyze the exchange of GDP for GTP to activate RhoA, a member of the Rho family of small GTPases that regulate a number of cell functions, including actin cytoskeletal organization, cell adhesion, and cell motility [14]. There are a number of receptor mechanisms that activate the Rho signaling pathway (directly or indirectly), including: stimulation of G protein-coupled receptors (LPA, ET-1) [15, 16], integrins (CTGF, Fibronectin) [17, 18], chemokines and growth factor receptors (TGFß-1) [19], many of which have an established role in the onset and progression of CKD.

The activation of RhoA stimulates the downstream effector Rho-associated coiled-coil protein kinase (ROCK), which phosphorylates LIM kinase (LIMK), myosin light chain (MLC), and MLC phosphatase (MLCP), subsequently impacting a number of cellular processes [20]. Phosphorylated LIMK, in turn phosphorylates cofilin (an actin binding protein) which plays an important role in the regulation of actin dynamics, leading to the depletion of G-actin pools, enhanced actin polymerization (F-actin) and can result in stress fiber formation (i.e., changes in cell shape), gene expression, and cell transformation (fibroblast to myofibroblast, EMT) [21]. Changes in actin dynamics (ratio G/F-actin pool) also impact serum response factor (SRF), a transcription factor that binds the serum response element (SRE) in a number of genes associated with cytoskeletal proteins (e.g., α-SMA), and promote myofibroblast activation, and synthesis of extracellular matrix proteins [14, 22]. ROCK activation by Rho also leads to phosphorylation of MLC and MLC phosphatase (inactivation) which can subsequently promote the development of stress fibers, impact cell contractility, and cell-cell contacts, including the regulation of tight junctions and the perijunctional actomyosin ring structure required for a polarized epithelial phenotype [23]. Thus, ARHGEF11 plays a central role in activating the Rho signaling cascade via a number of cell stimuli that can impact cytoskeletal structure that influence cell-cell contacts and promote cell transformation.

The present work established that *Arhgef11* is expressed at higher levels in the S (kidney injury) compared to Arhgef11-congenic and SHR (renal protective) starting before the onset of kidney injury (week 2). This pattern suggests that increased expression is regulated early at the genomic level (e.g. sequence variants in promoter region) as opposed to being the consequence of physiological differences. However, with time, *Arhgef11* expression becomes greater in the S, suggesting that progressive injury in the kidney acting through cytokines/chemokines (and other factors) lead to up-regulation of Rho-ROCK. The human biopsy findings support this mechanism as ARHGEF11 straining became more intense with progressive injury to the kidney.

*Arhgef11* knockdown cell lines were developed (HEK293 and NRK) to determine how changes in *Arhgef11* expression would impact the Rho-ROCK pathway and affect cell morphology. These cells demonstrated reduced RhoA activity, decreased activation of Rho-ROCK pathway (substantial decrease in the phosphorylated form of MLC and cofilin), and less stress fiber formation under baseline and serum free conditions. These findings are similar to work by others using different cell lines [23]. Genetic knockdown of *Arhgef11* and pharmacological inhibition of ROCK by fasudil (an inhibitor of ROCK) had similar impact on attenuating stress fiber formation.

Primary proximal tubule cells cultured from the S and Arhgef11-congenic kidney were studied as these cells capture the full extent of allelic variants between strains throughout the entire *Arhgef11* gene, including putative promoter region, exon (amino acid changes), and intron regions (potential transcript variant differences) [9]. Primary cells derived from the S exhibited increased expression of *Arhgef11*, increased RhoA activity, and up-regulation of Rho-ROCK signaling cascade similar to that observed in whole kidney [9]. The impact of TGFβ-1 on the primary cells were examined for two main reasons, (1) TGFβ-1 is a well-studied profibrogenic cytokine that plays an important role in the process of CKD and renal fibrosis [24]; and (2) TGFβ-1 is elevated in kidney of the S rat and anti- TGFβ-1 treatment[25] or gene knockout[26] has a renal protective effect in the S and other experimental models [27, 28]. PTC derived from the S kidney were more prone to EMT, a hallmark feature of the development of renal fibrosis, compared to Arhgef11-congenic derived cells.

The ability of primary PTC to uptake albumin was examined between S and Arhgef11-congenic cells as the inability of proximal tubules to re-uptake filtered protein and/or become overloaded by albumin can lead to proinflammatory and profibrotic effects that contribute to the development of tubulointerstitial injury [29, 30]. In particular, albumin overload in PTC has been shown to induce the secretion of a number of inflammatory factors including chemokine (C-C motif) ligand 5, monocyte chemotactic protein-1, interleukin 6, and TGFβ-1 [31]. The PTC derived from the S demonstrated decreased ability to uptake FITC-albumin, while Arhgef11-congenic cells demonstrated a significant improvement. The FHH was utilized as a negative control for protein uptake as it is known to have a genetic defect (Rab38) that is responsible for impairment in the tubular re-uptake of filtered protein [32]. The *in vitro* findings were supported by *in vivo* assessment of re-uptake of filtered FITC-albumin. While both the S and Arhgef11-congenic exhibit similar glomerular permeability[9] (i.e. the amount of protein in the ultrafiltrate is similar), the Arhgef11-congenic demonstrated greater re-uptake in proximal tubule (increased tubular/punctate fluorescence), which ultimately resulted in less proteinuria compared to the S. The SHR demonstrated significantly lower fluorescence (re-uptake of FITC-albumin) because little protein makes it through to the ultrafiltrate, which is then actively taken up by the proximal tubules.

**Fig 10:**
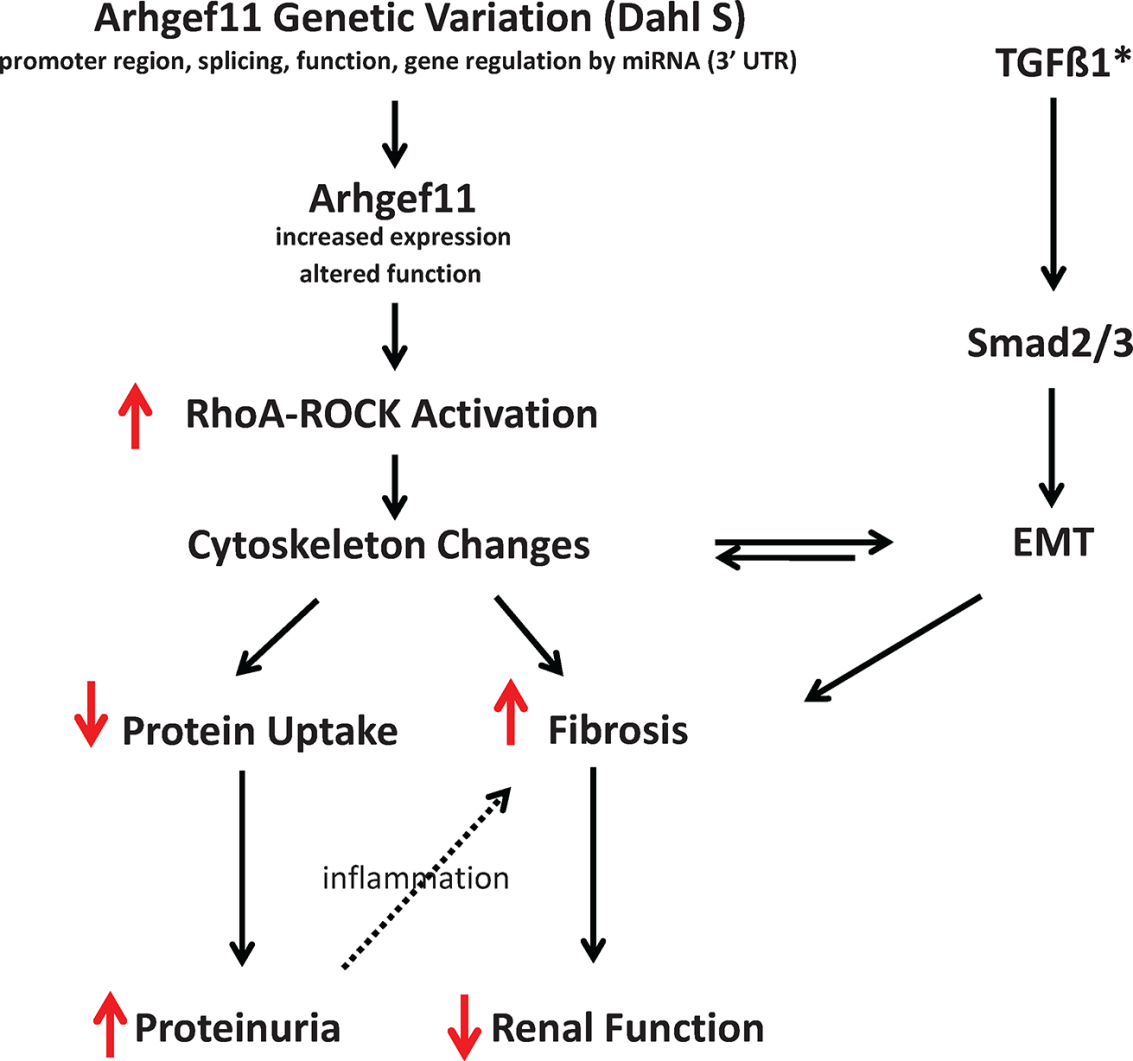
Overview of proposed role of the Arhgef11 in promoting renal injury and decline in renal function in the Dahl S. Genetic variants in the Dahl S form of *Arhgef11* (via increased expression and/or functional changes that increase GTP to GDP exchange) lead to chronic activation of RhoA-ROCK pathway. The increased activation in RhoA-ROCK pathway promote cytoskeletal changes that impairs the ability of tubules to re-uptake filtered protein, which leads to proteinuria. These cytoskeletal changes, in the context of TGFß-1, and other inflammatory factors (other susceptibility loci) leads to disruption of cell-cell contacts, predisposition to epithelial mesenchymal transition (EMT), and culminates in tubulointerstitial injury/fibrosis and reduced renal function. *, in the Dahl S model, TGFß-1 is elevated due to genetic predisposition to develop kidney injury. While the Arhgeh11-congenic model exhibits some renal protection, it still contains several other susceptibility loci that promote kidney injury and increased cytokines/chemokines.

Considering all the data, the major findings of this study are: (1) increased expression of *Arhgef11* occurs early in the kidney and both precedes and correlates with progression of kidney injury and reduced kidney function in the S rat; (2) *Arhgef11* expression in human kidney biopsies was associated with degree of injury (e.g. increased expression = increased injury) regardless of the underlying kidney pathology; (3) stress-fiber (F-actin) formation was significantly attenuated in *Arhgef11* knockdown cells under baseline and serum-free conditions compared to control; (4) primary proximal tubules cells derived from S kidney exhibit up-regulation of Rho-ROCK, increased Rho activity, and greater susceptibility toward EMT under baseline and with TGFß-1 treatment versus theArhgef11-congenic; and (6) S rat derived primary proximal cells (versus Arhgef11-congenic) exhibit decreased uptake of FITC-albumin *in vitro*, which is consistent with decreased ability to re-uptake filtered protein *in vivo*. Based on these findings we propose that allelic variant in the S form of *Arhgef11* (compared to SHR) leads to increased expression and/or activity of *Arhgef11*, chronic activation of the RhoA-ROCK pathways which promotes cytoskeletal changes that impact proximal tubular function, and in the context of other susceptibility factors (e.g. other genetic factors in the S), enhance EMT and development of tubuloinsterstial fibrosis to promote decline in renal function (**Fig 10**).

Evidence that genetic variants in human ARHGEF11 is associated with CKD is limited. However, in a previous study, we identified a significant association between SNPs around human ARHGEF11 and eGFR (estimated glomerular filtration rate) using the Candidate Gene Association Resource (CARe) population (n = 23,247) [9]. Polymorphisms in ROCK has been associated with risk of hypertension in HYPGENE study [33]. Irrespective of whether genetic polymorphism in ARHGEF11 or other Rho-ROCK genes are ultimately causative to CKD, pharmacological inhibition of this pathway via ROCK (fasudil and Y-27632) has been demonstrated to be effective at improving proteinuria, glomerulosclerosis, and fibrosis in experimental animal models [34–36]. There have been several other human studies that have identified an association between ARHGEF11 and metabolic, inflammatory diseases, and psychiatric disorders, such as impaired glucose tolerance and diabetes[37, 38], susceptibility to intracranial aneurysm[39], and schizophrenia [40]. There is no experimental evidence to support a potential mechanism between ARHGEF11 and these disease processes, but it is likely to function through the Rho-ROCK pathways as ARHGEF11 plays a central role in activating this pathway.

While this study focused on the impact of Arhgef11-Rho signaling in tubular cells due to the more prominent role in EMT and promoting fibrosis in our model; chronic stimulation of this pathway in other kidney cell types including mesangial, glomerular endothelium, and renal vasculature (vascular smooth muscle cells) could contribute to the pathophysiology of CKD in the S model. Given that there are a number of genetic loci have been associated with kidney injury in the S rat (compared with SHR), it is also possible that other genes directly/or indirectly contribute to this *Arhgef11*-Rho mechanism of injury. For example, a strong interaction between RNO2 (*Arhgef11*) and RNO8 has been observed, which itself exhibits an independent impact on kidney injury and function (both by linkage analysis[13] and congenic strain analysis[10]). The genomic locus contains paracingulin/cingulin-like 1 (*Cgnl1*), which is known to interact with ARHGEF2 (similar to ARHGEF11) [41]. Paracingulin is detected at high-levels in the kidney, particularly in tight junctions and apical junctions and depletion of paracingulin (*in vitro*) results in increased RhoA activity, activating a number of pathways [41, 42].

In summary, we have identified a gene involved in CKD by positional cloning and established a potential mechanism supporting the functional significance of genetic variants in *Arhgef11* in promoting changes in cell morphology (Rho-Rock and/or EMT) and function of tubule cells that works in concert with other susceptibility factors (e.g. TGFβ-1) to promote progressive decline in kidney function. The elucidation of molecular mechanisms of CKD in the Dahl S model will not only provide fundamental insight into the genetic basis of hypertension related kidney disease and genetic interactions, but will provide an opportunity to identify more specific drug targets for therapy in humans.

## Material and Methods

### Animals and Human Biopsies

All animal experiments were approved by the Institutional Animal Care and Use Committee at the University of Mississippi Medical Center (UMMC). The Dahl salt-sensitive (S), Arhgef11-congenic [S.SHR(2)X39], spontaneously hypertensive rat (SHR), and fawn hooded hypertensive (FHH) strains are maintained at our institutional animal facility. The collection of human kidney biopsies were approved by the Institutional Review Board at UMMC.

#### Measurement of blood pressure, proteinuria, and renal function

At 4 weeks of age, groups of age matched male S, Arhgef11-congenic, and SHR animals (n = 6–20 per group) were weaned onto low-salt diet (0.3% NaCl; TD7034; Harlan Teklad, Madison, WI). Subsequently, week 4 animals were assessed for proteinuria (24-hr urine collections), creatinine clearance, and terminal blood pressure measurement as previously described [43, 44]. A separate group of animals were raised to week 24 for determination of proteinuria, creatinine clearance, and blood pressure (n = 6 per group).

#### Filtration of FITC-labeled albumin in the Kidney

FITC-labeled albumin (20mg/kg; Sigma) was infused in S, Arhgef11-congenic and SHR (n = 5–6 per group) at a rate of 100 ul/min for 15 min. Subsequently, kidneys were collected, fixed in a 10% buffered formalin solution, processed and sectioned, and counterstained with Alcian blue. Images were obtained using a Nikon 55i fluorescence microscope equipped with DS-Fi1 5-Meg Color C digital camera (Nikon, Melville, NY). Thresholding was used to measure the degree of fluorescence in the kidney from each strain (n = 2 sections per kidney and 10–15 images per section) using Nikon Elements software.

#### Histology of rat kidney and immunohistochemistry of human kidney biopsies

Kidneys were fixed in 10% buffered formalin, embedded in paraffin, cut into 4-μm sections and stained with hematoxylin and eosin (H&E) and/or Masson’s trichrome (n = 5–6 per group/time point). ARHGEF11 localization in human kidney biopsy was assessed by immunohistochemistry on unstained sections using primary antibodies directed at ARHGEF11 (Novus Biologicals, Co) and detected by DAB (Ultravision LPValue Detection System, Thermo Scientific). Slides were counterstained with methyl green. Images were captured using Nikon 55i microscope with DS-Fi1 5-Meg Color C digital camera (Nikon, Melville, NY).

### Cell lines, Primary Culture, and Lentiviral Knockdown

#### Cell-Lines

HEK293T/17 human embryonic kidney cell line (CRL-11268) and NRK (Normal Rat Kidney, CRL-1571) cells were obtained from ATCC (Manassas, VA). Both cell lines were cultured according to ATCC protocols and maintained in Dulbecco’s modified Eagle’s medium (DMEM) supplemented with 10% fetal bovine serum and antibiotic and antimycotic solution (Life Technologies, CA) at 37°C in 5% CO2.

#### Lentiviral knockdown of Arhgef11 using shRNA/stably transduced cell-lines

Four short hairpin RNA (shRNA1-4), targeting different regions of *Arhgef11* were cloned into Lenti-Pac HIV Expression Packaging Kit (GeneCopoeia, MD). All four shRNA were specific to rat and one (of four) was a perfect match with human Arhgef11 (**S2 Fig**). Plasmids containing each shRNA/GFP or scrambled control and packaging plasmid were co-transfected into HEK293T/17 or NRK (~ 90% confluent) at ratio of 2:1:1 per manufacture instructions. Cells for GFP control plates were checked regularly under the fluorescent microscope. Culture medium containing virus was collected 48–72 hrs after transfection, harvested, and concentrated 10-50-fold using Lenti-Pac Lentivirus Concentration Solution (GeneCopoeia, MD). Virus titers were determined using H1299 cells by fluorescence analysis. 10^9 IU/ml virus was stored in 1 ml aliquots at −80 °C until cell transduction. HEK293T and NRK were transduced with concentrated lentiviral stocks in the presence of 8 μg/ml polybrene (Sigma-Aldrich, MO). Stably transduced cells were selected using 2ug/ml puromycin (Life Technologies l) for 3–5 days and purity of transduced cells was evaluated by GFP fluorescence microscopy.

#### Proximal tubule isolation and primary culture

Animals were euthanized under isoflurane gas. Kidneys were removed, immediately placed into ice cold HBSS (without Ca^2+^ or Mg^2+^), and transferred to biological safety hood where the cortex was carefully dissected away from the medulla. The cortex was finely minced and transferred into a 15 ml conical tube containing 7 ml of pre-warmed HBSS with DNAsae (20μg/ml), Collagenase (1.4mg/ml), Trypsin inhibitor (0.033mg/ml), and gently mixed 37°C for 20 minutes. Fresh collagenase solution until tissue was digested (2–3 times). The digested material was placed on a >125um sieve and gently pushed through sieve with a sterile plunger. The sieve was washed several time in HBSS (with Ca^2+^ or Mg^2+^) and the flow through discarded containing cellular debris and glomeruli. The material on the top of sieve was collected using a serological pipette, placed in 50ml conical tube, and centrifuged on low speed (300Xg) for 5 minutes. The supernatant was decanted and the material was resuspended in DMEM/F12 media (containing 0.05% BSA, fraction, 1X Insulin/Transferrin/Selenium solution, 1X Penicillin/ Streptomycin, 50 nM Hydrocortisone) and seeded into 6-well collagen I coated plates (Becton Dickinson, MA) at ~20mg/well (3.5cm^2^) in a total of 3mls. Media was changed to 2mls after first 24 hours then every 2 days after for up to 8 days. Visual inspection of the material by microscopy demonstrated that it was composed primary of small tubule fragments (>95%) and small number of glomeruli.

#### Immunofluorescence

Cells for immunofluorescence were seeded into 8-well chamber slides (2000–5000 cells) and incubated at 37°C, 5% CO2 for at least 12 hours for cell adherence. Chamber slides were washed 3 times in 400ul PBS, fixed for 30 min with 400ul 4% paraformaldehyde, and permeabilized in 0.2% Triton X / PBS for 20 minutes at room temperature (RT). Cells were blocked in 5% goat serum/PBS for 1 hour, incubated with primary antibody at optimal concentration in 2% goat serum/PBS for 2 hours at RT. Appropriate secondary antibody [e.g. anti-goat-Alexa 546 and anti-rabbit-Alexa 488 (Invitrogen)] in 2% goat serum / PBS was added to each well and incubated at RT for 60 minutes. 100μl of Vectashield (Vector Lab) was added and cover slip was added. Images were captured using Nikon 55i microscope with DS-Fi1 5-Meg Color C digital camera (Nikon, Melville, NY) and analyzed using Nis-Elements image analysis software (version 3.03, Nikon Instruments Inc., Melville, NY).

### Activity and Cell Assays

#### RhoA activity assay

RhoA activity was assessed using G-LISA RhoA activation assay kit (Cytoskeleton, Inc. Denver CO). Cells were grown as described above for cell-lines and/or primary cells and processed per manufacturer instructions. Protein concentrations were normalized across samples for each assay. Absorbance was measured at OD490 using Synergy BioTek plate reader.

#### FITC-Albumin Uptake Assay

PTC cells were cultured in 12 well plates (> 60% confluence) and assayed at 5 days post-seeding. Cells were washed 3 times with PBS, and incubated with either FITC-Albumin or FITC-Dextran [10ug/ml PBS (with HEPS 10nM)] for 5, 10, 15, 30, or 60 minutes. At the conclusion of each incubation period, cells were rinsed multiples times to remove unbound FITC. Cells were collected in RIPA buffer and FITC fluorescence (EM: 485/20, and 528/20) was measured using Synergy BioTek plate reader using a standard curve. Albumin uptake was normalized to total protein (mg/g protein).

### Real-Time PCR and Western Blot Analysis

#### Real-Time PCR

RNA was isolated using TRIzol and Invitrogen PureLink Kit (Life Technologies) and evaluated for quality and integrity (Bio-Rad Experion System) as previously described [43, 44]. RNA was reverse-transcribed to cDNA using iScript cDNA Synthesis Kit and real-time PCR (Bio-Rad) was performed using SsoFast EvaGreen Supermix (Bio-Rad). Gene expression was evaluated using SYBR-green dye chemistry on a Bio-Rad CFX96 platform (n = 6).

#### Western blot analysis

Tissue and cells homogenates were prepared in RIPA lysis buffer (Santa Cruz Biotechnology) and western blot prepared using standard methods [43]. Protein concentration was determined using Bio-Rad protein assay kit (Bio-Rad). Blots were probed with Rho-ROCK pathway antibodies (Arhgef11, RhoA, Rock1, Limk1, and Cofilin, TGFβ-1, E-cadherin and N-Cadherin) and appropriate secondary antibody and imaged using a Pierce ECL Substrate on ChemiDoc XRS+ System and software (Bio-Rad).

### Statistical Analysis

Statistical analyses were performed using GraphPad Prism 6.0 software (San Diego, California, USA). Data was analyzed using independent t-test or one-way ANOVA followed by either Dunnett’s or Bonferroni. Results were expressed as the mean ± SD for all cell-culture based work and mean +SE for physiological studies. A p< 0.05 was considered to be statistically significant.

## Supporting Information

S1 Fig Overview of the experimental evidence supporting *Arhgef11* as candidate gene for kidney injury in Dahl S rat. The high-resolution localization of *Arhgef11* was achieved by linkage, congenic strain analysis, comparative genomics, and sequencing. The black bar on the left of the ideogram shows the 95% confidence interval (CI) for the quantitative trait loci (QTL) from original linkage analysis [13] and 95% CI from second larger population, n = 993 (open bar)[12] and The red box denotes the current refinement of kidney injury locus to a <375 kb (box on ideogram) [9] and the location of SHR genome on the S genetic background (Arhgef11-congenic). The nature and type of sequence variation (coding/promoter), increased expression, and biological role suggest that *Arhgef11* likely plays an important role in kidney injury and decline in kidney function.
(TIFF)

S2 Fig Schematic diagram of location of sequence differences between the S and SHR allele of *Arhgef11*. Each type of genetic variation has the potential to impact gene expression (transcription factor binding), protein function (amino acid changes), and/or transcript variants (RNA splicing). There are a number of allelic differences (SNP or INDEL) or combination of variants in the S form of Arhgef11 (compared to SHR allele) that could explain increased expression and activity.
(TIFF)

S3 Fig Selection of lentiviral shRNA to generate *Arhgef11* knockdown in NRK and HEK293T cells. (**A**) Stably transduced cells were generated using lentiviral constructs containing shRNA1-4 (perfect match with rat) in NRK cells and shRNA1 and 3 (mismatch and perfect match) in HEK293T. (**B**) Schematic of location of shRNA1-4 targets in rat and human *Arhgef11* genes. (**C**) Efficiency of *Arhgef11* knockdown and subsequent impact on RhoA by real-time PCR. Stably transduced shRNA1 had no impact on Arhgef11, while shRNA2 has slight, but significant knockdown of Arhgef11. Stably transduced shRNA3-4 demonstrated a 60–80% decrease in *Arhgef11* expression both NRK and HEK293T cells. However, stably transduced shRNA3 NRK and HEK293 cells were used for subsequent experiments (i.e. perfect match of shRNA3 to both rat and human). n = 4–6 independent samples, *p<0.05 versus LVC. Error bars are ±SD.
(TIF)

S4 Fig Analysis of the Rho-ROCK signaling pathway and RhoA activity in stably transduced *Arhgef11* knockdown NRK cell lines. Cell-culture experiments were performed under several experimental conditions: 1) control (DMEM medium); 2) fasudil (F) (10μg/ml) treated for 4hrs; 3) serum free (SF) for 24 hours, or 4) serum free for 24 hrs + fasudil treated for 4 hours (SF+F). (**A**) Real time PCR of *Arhgef11*, *RhoA*, *Rock1*, *MLC* and *Cofilin* of NRK-shRNA3 compared to LVC (scrambled control). (**B**) RhoA activity between LVC and NRK-shRNA3 under each experimental condition. (**C**) Western analysis of Rho-Rock pathway in LVC and NRK-shRNA3 between control and fasudil treatment. Similar results were observed for LVC and HEK293-shRNA3 (**Fig 4**). n = 6 independent samples per group/treatment, *p<0.05 versus LVC; †p<0.05 versus LVC+F or LVC+SF. Error bars are ±SD.
(TIF)

S5 Fig Densitometry measurement of Arhgef11-Rho-Rock proteins between LVC and HEK293-shRNA3 between control, fasudil treatment, and serum free conditions. (**A**) Densitometry measurement of Rho-Rock pathway in LVC and HEK293-shRNA3 between control and fasudil treatment; and (**D**) Densitometry measurement of Rho-Rock pathway in LVC and HEK293-shRNA3 between control and serum free conditions. n = 6 independent samples per group/treatment, *p<0.05 versus LVC. Error bars are ±SD.
(TIF)

S6 Fig RhoA Activity in primary proximal tubule cell (PTC) treated with TGFß-1. Primary PTC cells (day 5, >60% confluent) were grown from S and Arhgef11-congenic kidney (at 4 weeks of age) and treated with TGFß-1 (10 ng/ml) for 48 hrs. (**A**) Western analysis and densitometry of Arhgef11 and RhoA signaling pathway. (**B**) RhoA activity. n = 6 independent samples, *p<0.05 versus S; †p<0.05 versus S and S+ TGFß-1. Error bars are ±SD.
(TIF)
