# Supplementary figures and images for: Allelic Variants in Arhgef11 via the Rho-Rock Pathway Are Linked to Epithelial–Mesenchymal Transition and Contributes to Kidney Injury in the Dahl Salt-Sensitive Rat
Source: PLoS One. 2015 Jul 14;10(7):e0132553. doi: 10.1371/journal.pone.0132553 (PMC4501567; doi:10.1371/journal.pone.0132553)

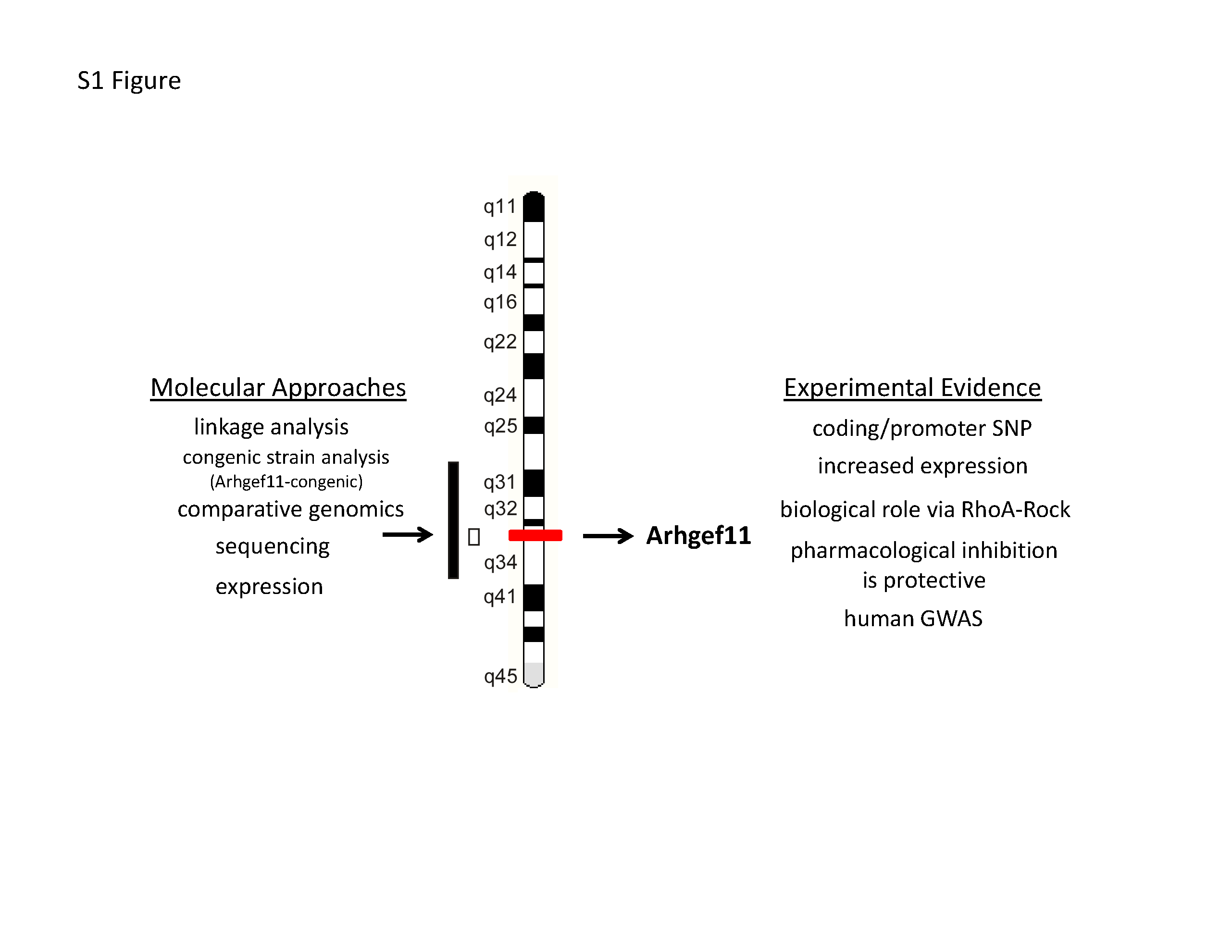

Supplement: S1 Fig — The high-resolution localization of Arhgef11 was achieved by linkage, congenic strain analysis, comparative genomics, and sequencing. The black bar on the left of the ideogram shows the 95% confidence interval (CI) for the quantitative trait loci (QTL) from original linkage analysis [13] and 95% CI from second larger population, n = 993 (open bar)[12] and The red box denotes the current refinement of kidney injury locus to a <375 kb (box on ideogram) [9] and the location of SHR genome on the S genetic background (Arhgef11-congenic). The nature and type of sequence variation (coding/promoter), increased expression, and biological role suggest that Arhgef11 likely plays an important role in kidney injury and decline in kidney function. (TIFF) [file pone.0132553.s001.tiff]

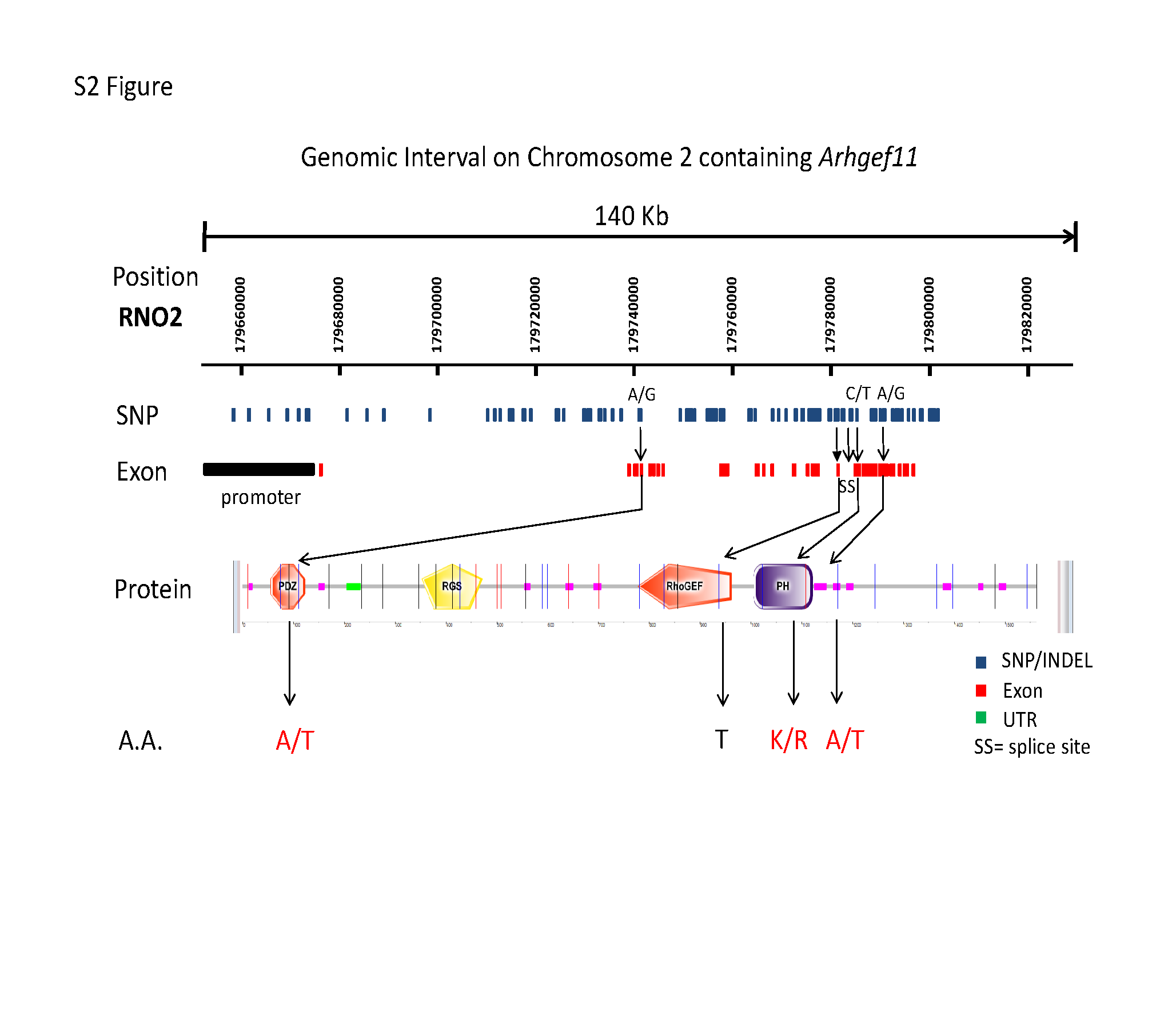

Supplement: S2 Fig — Each type of genetic variation has the potential to impact gene expression (transcription factor binding), protein function (amino acid changes), and/or transcript variants (RNA splicing). There are a number of allelic differences (SNP or INDEL) or combination of variants in the S form of Arhgef11 (compared to SHR allele) that could explain increased expression and activity. (TIFF) [file pone.0132553.s002.tiff]

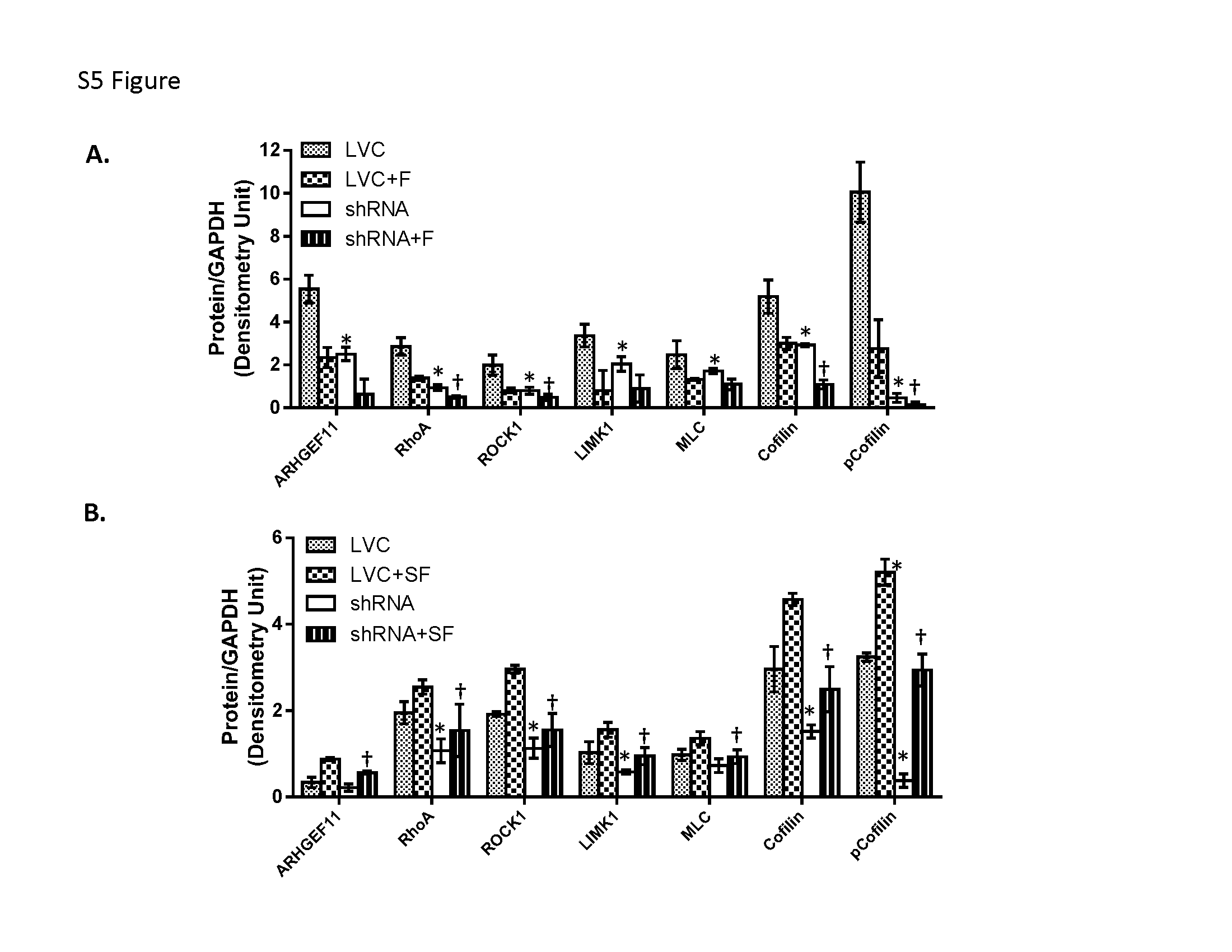

Supplement: S5 Fig — (A) Densitometry measurement of Rho-Rock pathway in LVC and HEK293-shRNA3 between control and fasudil treatment; and (D) Densitometry measurement of Rho-Rock pathway in LVC and HEK293-shRNA3 between control and serum free conditions. n = 6 independent samples per group/treatment, *p<0.05 versus LVC. Error bars are ±SD. (TIF) [file pone.0132553.s005.tif]

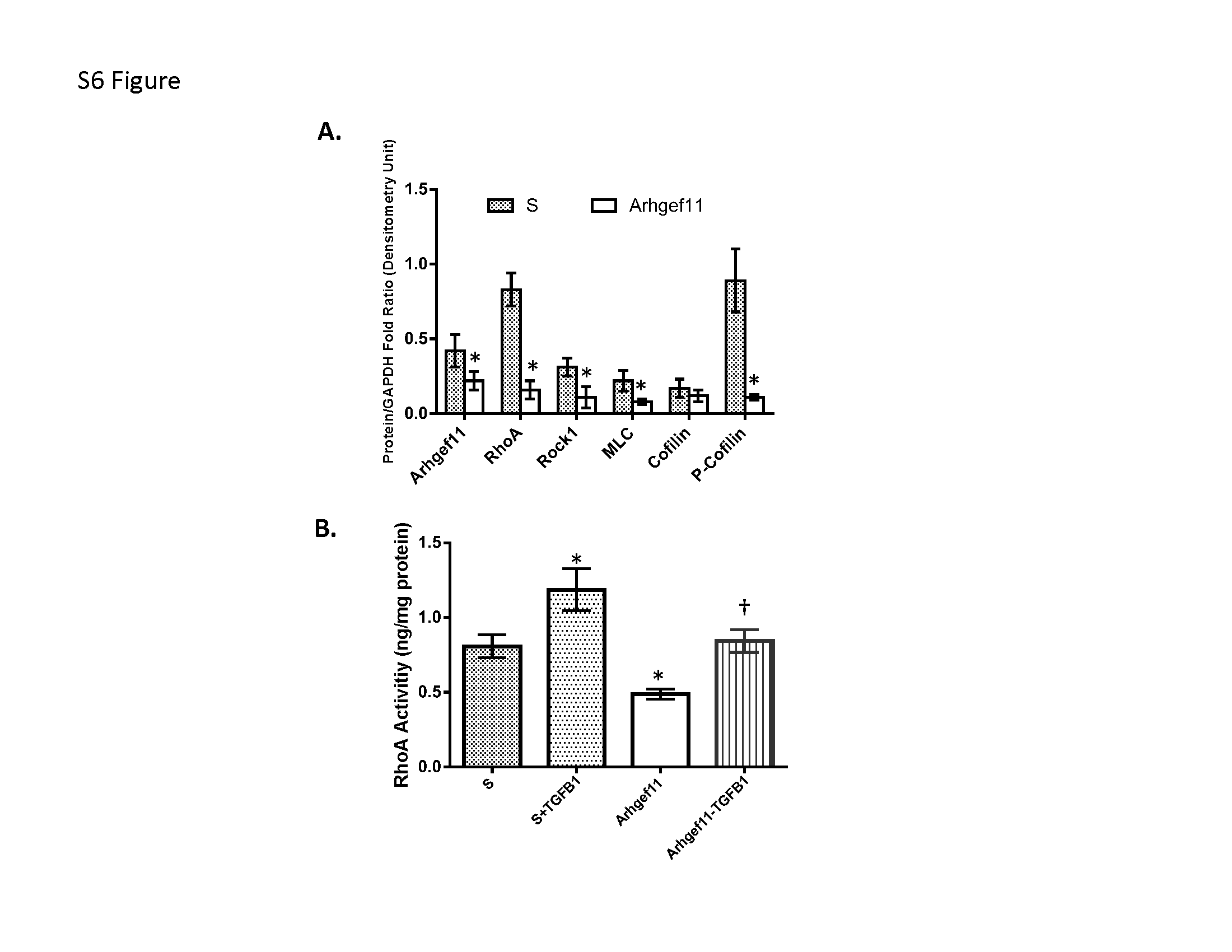

Supplement: S6 Fig — Primary PTC cells (day 5, >60% confluent) were grown from S and Arhgef11-congenic kidney (at 4 weeks of age) and treated with TGFß-1 (10 ng/ml) for 48 hrs. (A) Western analysis and densitometry of Arhgef11 and RhoA signaling pathway. (B) RhoA activity. n = 6 independent samples, *p<0.05 versus S; †p<0.05 versus S and S+ TGFß-1. Error bars are ±SD. (TIF) [file pone.0132553.s006.tif]
